# Small RNA profiles in soybean primary root tips under water deficit

**DOI:** 10.1186/s12918-016-0374-0

**Published:** 2016-12-05

**Authors:** Yun Zheng, Vandana Hivrale, Xiaotuo Zhang, Babu Valliyodan, Christine Lelandais-Brière, Andrew D. Farmer, Gregory D. May, Martin Crespi, Henry T. Nguyen, Ramanjulu Sunkar

**Affiliations:** 1 Faculty of Information Engineering and Automation, Kunming University of Science and Technology, Kunming, Yunnan 650500 China; 2 Department of Biochemistry and Molecular Biology, Oklahoma State University, Stillwater, OK 74078 USA; 3 National Center for Soybean Biotechnology and Division of Plant Sciences, University of Missouri, Columbia, MO 65211 USA; 4 Institut of Plant Sciences Paris-Saclay (IPS2), CNRS, INRA, University of “Paris-Sud”, Batiment 630, 91405 Orsay, France; 5 Institut of Plant Sciences Paris-Saclay (IPS2), CNRS, INRA, University of “Paris-Diderot”, Sorbonne Paris-Cité, 91405 Orsay,, Paris, France; 6 National Center for Genome Resources, Santa Fe, New Mexico, NM 87505 USA; 7 Present address: Pioneer Hi-Bred International, Inc, Johnston, IA 50131 USA

**Keywords:** Drought, miRNAs, Primary root tips, Post-transcriptional gene regulation, Soybean

## Abstract

**Background:**

Soybean (*Glycine max*) production is significantly hampered by frequent droughts in many regions of the world including the United States. Identifying microRNA (miRNA)-controlled posttranscriptional gene regulation under drought will enhance our understanding of molecular basis of drought tolerance in this important cash crop. Indeed, miRNA profiles in soybean exposed to drought were studied but not from the primary root tips, which is not only a main zone of water uptake but also critical for water stress sensing and signaling.

**Methods:**

Here we report miRNA profiles specifically from well-watered and water-stressed primary root tips (0 to 8 mm from the root apex) of soybean. Small RNA sequencing confirmed the expression of vastly diverse miRNA (303 individual miRNAs) population, and, importantly several conserved miRNAs were abundantly expressed in primary root tips.

**Results:**

Notably, 12 highly conserved miRNA families were differentially regulated in response to water-deficit; six were upregulated while six others were downregulated at least by one fold (log2) change. Differentially regulated soybean miRNAs are targeting genes include auxin response factors, Cu/Zn Superoxide dismutases, laccases and plantacyanin and several others.

**Conclusions:**

These results highlighted the importance of miRNAs in primary root tips both under control and water-deficit conditions; under control conditions, miRNAs could be important for cell division, cell elongation and maintenance of the root apical meristem activity including quiescent centre whereas under water stress differentially regulated miRNAs could decrease auxin signaling and oxidative stress as well as other metabolic processes that save energy and water.

**Electronic supplementary material:**

The online version of this article (doi:10.1186/s12918-016-0374-0) contains supplementary material, which is available to authorized users.

## Background

MicroRNAs are known to regulate the abundances of messenger RNAs that have complete or partial complementarity and inhibit protein synthesis from those targets [1, 2]. MicroRNA-guided gene regulation is vital for almost all aspects of plant growth and development [2–4] as well as biotic and abiotic stress responses [5].

Soybean (*Glycine max*) is a major leguminous crop cultivated for vegetable oil and protein source for human consumption and animal feed, besides being used in aquaculture and poultry [6]. In the recent years, drought has significantly affected soybean yields throughout the world including the United States. Root system is most important organ with respect to drought responses; therefore, analyzing miRNAs could reveal critical processes important for drought tolerance in soybean. Within the root system, well-developed or older or matured root zone is less active not only metabolically but also with respect to its role in signalling, therefore analyzing primary root tip is critically important not only under water deficit conditions but also under normal conditions.

A recent study profiled small RNAs from diverse soybean tissues [7] but not from the primary root tips. Similarly, miRNAs were profiled from the entire root system of soybean exposed to drought or water stress, but not in the primary root tips [8, 9]. These published studies found similar as well as distinct regulations with respect to the responsiveness of various miRNAs, which could be attributed to differences in genotypes, treatments, duration or other experimental variables.

Compared to many soybean genotypes, the soybean cv. Magellan, which was used in the current study, was relatively drought-tolerant [10, 11]. Interestingly, on the basis of differential responses with respect to elongation rates during stress, primary root tip region can be divided into two regions (Region 1 [0–4 mm from the apex, including the root cap] and Region 2 [4–8 mm from the apex]) in this genotype [10]. Under water stress, Region 1 maintains elongation rates but Region 2 shows progressive deceleration, however, under well-watered conditions, only Region 2 maintains maximum elongation rates [10, 11]. Well-developed or older or matured root zone is less active not only metabolically but also with respect to its role in signalling, therefore analyzing molecular responses in primary root tip is critically important under water deficit conditions. Previously, proteome [10] as well as transcriptome [12] changes in response to drought have been analyzed in soybean primary root tips. However, post-transcriptional gene regulation directed by miRNAs in this region of the soybean roots has not been investigated so far. Studying miRNA-guided gene regulations is a rational link that could help bridge the gap between transcriptome and proteome analysis in this important organ of the plant system. Therefore, here we report miRNA profiles to further our understanding of drought tolerance mechanisms in the primary root tips of a tolerant soybean genotype.

## Methods

### Stress treatments, collection of root tips, RNA isolations and sequencing

The soybean seedlings (Magellan genotype) growth and treatments were conducted as reported previously [10]. In brief, seedlings with primary roots approximately 15 mm in length were transplanted against the interior surface of Plexiglas cylinders (14.5 cm diameter) filled with a 1:1 (v/v) mixture of vermiculite (no. 2A, Therm-ORock East Inc., New Eagle, PA, USA) and Turface (Profile Products LLC, Buffalo Grove, IL, USA) at water potentials of −0.1 MPa (well-watered control, moistened to the drip point) or −1.6 MPa (water stress), which were obtained by thorough mixing with different volumes of 5 mm CaCl_2_ + 5 mm Ca(NO_3_)_2_ solution. Primary root tip region (0–8 mm) was collected after 48 h of treatment along with the control samples in two replicates and total RNA was isolated and used for constructing small RNA libraries. Root tip water stress level was assessed by measuring water potential of the growing media using an isopiestic thermocouple psychrometer. The obtained small RNA libraries were sequenced using Illumina HiSeq 2000 equipments by following the corresponding protocols. Two replicate libraries per treatment (control and water-stressed) were constructed and sequenced. The obtained small RNA profiles had been deposited into NCBI SRA database under the series accession number SRP083572.

### Sequence analysis, identification of conserved and novel miRNAs as well as drought-responsive miRNAs

The extracted small RNA reads were screened against ribosomal and transfer RNAs, snRNAs and snoRNAs and exact matches to these RNA categories were discarded. Similarly, reads mapped to the transposons and protein-coding mRNAs were also discarded. Also that small RNAs shorter than 18 nucleotide (nt) and longer than 28 nt were discarded. The remaining reads were mapped to the miRBase to identify conserved and known miRNA homologs. The reads that could not belong to any of the above mentioned RNA categories were mapped to the genome in order to identify potential novel miRNAs, whose identification strictly followed the guidelines of Meyers et al. [13]. In brief, we mapped novel small RNAs to the genome and isolated the precursor region, which is about 150 nt both downstream and upstream to the mapped region and predicted fold-back structure for the precursor. If a fold-back structure could be predicted for the precursor region, then we searched for the presence of miRNA* reads in our small RNA libraries that is complementary to the potential novel miRNA.

For calculating the raw abundance of individual miRNAs within a family, the unique reads were used. Similarly, the raw abundance of all members were pooled to count the abundances of miRNA families. Abundances for each miRNA/miRNA family were normalized to Reads per 10 million (RPTM). For identification of drought-responsive miRNAs, fold (log2) change values were calculated. edgeR [14] was used to identify differentially regulated miRNAs and miRNA families in the treated primary root tips.

### Target prediction of miRNAs and tasiRNAs

The targets of miRNAs and tasiRNAs were predicted using the HitSensor algorithm [15]. Targets with less than 4 mismatches were kept for further analysis. The enrichment analysis for targets of some highly expressed deregulated miRNAs were performed with agriGO [16].

## Results

### Deep sequencing of small RNAs from soybean root tips exposed to drought

**Fig. 1:**
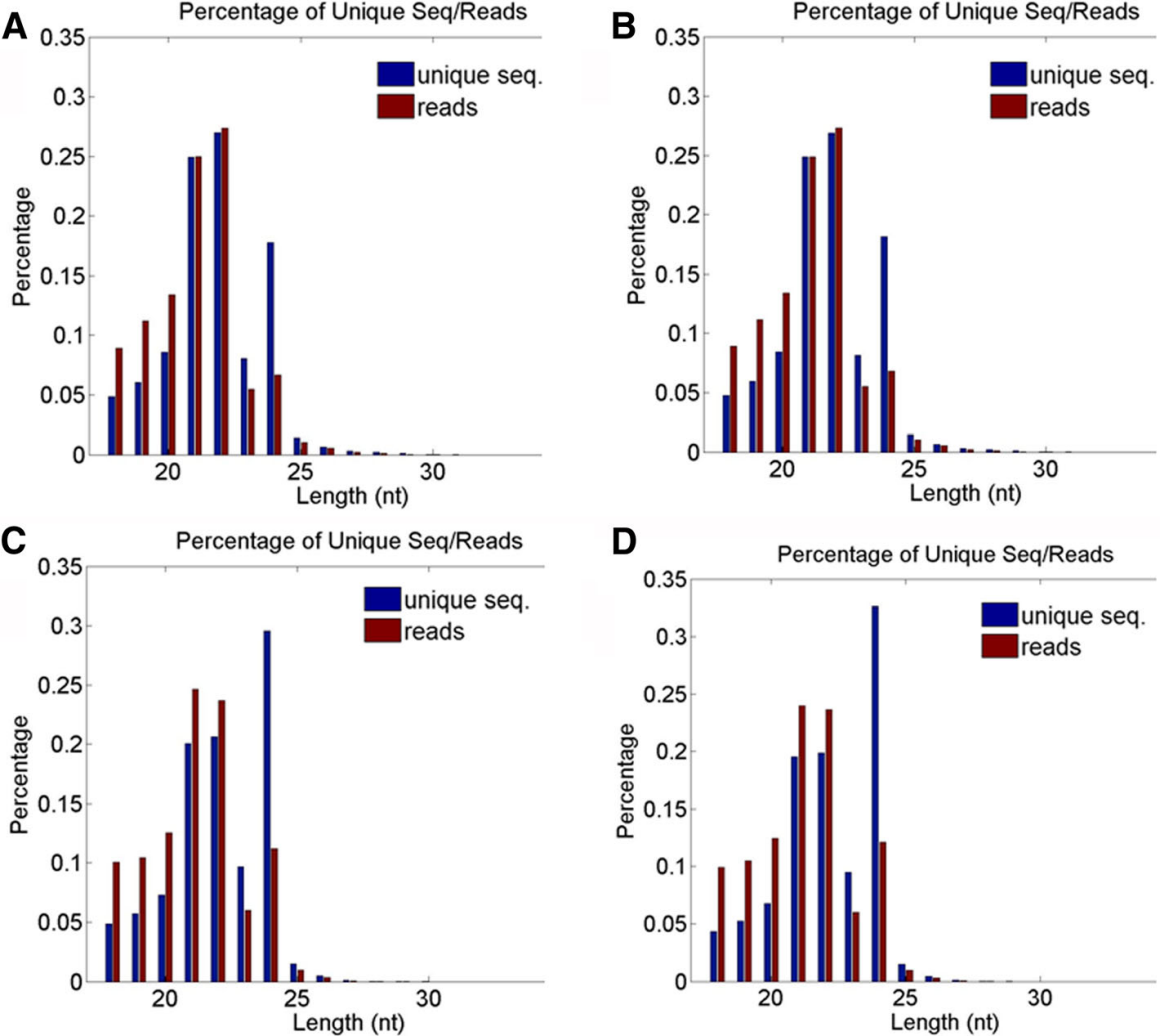
Total and Unique small RNA length vs abundances was plotted as percent of reads from 4 different small RNA libraries; **a** and **b** are two control libraries (control-1 and control-2) **c** and **d** are two water-stressed libraries (WS-1 and WS-2)

Two replicate small RNA libraries were constructed and sequenced from primary root tips of control and water deficit-exposed soybean seedlings. Each of these libraries (control and treated) from primary root tips yielded approximately 2 million reads. Overall, 7.5 million total reads represented by 1.8 million unique reads were obtained from these four small RNA libraries from primary root tips (Table 1). Small RNA length distribution versus abundances revealed a peak each at 21 and 24 nt size classes, resembling typical plant small RNA populations (Fig. 1). On the basis of their sequence similarity with the deposited miRNAs at the miRBase, we have identified 303 miRNA homologs (Additional file 1), which could be clustered into 171 families (Additional file 2).

**Table 1:** Summary of small RNA reads obtained from the primary root tips exposed to water deficit or control plants

	Control-1		Control-2		Water stress-1		Water stress-2	
	Total reads	Unique reads	Total reads	Unique reads	Total reads	Unique reads	Total reads	Unique reads
mRNAs	155,922	60,688	163,797	63,426	104,248	39,444	144,385	519,42
miRBase	415,510	1967	435,726	2019	313,001	1591	430,726	1890
Noncoding RNA	244,891	10,514	256,746	10,703	204,979	8317	274,264	9421
Repeats	463,369	28,499	490,924	29,226	325,516	22,815	451,881	26,078
Genome	1,664,319	416,494	1,756,718	436,528	1,222,109	333,042	1,717,876	453,431
Total	2,011,156	552,990	2,121,444	577,244	1,466,922	456,125	2,029,346	596,687

### Analysis of miRNA abundances in primary root tips of soybean

MicroRNAs have emerged as critical regulators of gene expression which is important for normal growth and development of leaves, roots, flowers and other morphological traits in plants. Primary root apex is critical for root growth and development under normal conditions. To assess the importance of miRNAs in this root zone of soybean, we measured their levels. Indeed, the abundances of different miRNA families varied greatly in the primary root tips. On the basis of their normalized abundances, miRNA families were divided into high (100,000 RPTM and above), moderate (10,000 to 100,000 RPTM), low (1000 to 10,000 RPTM) and extremely low (1 to 1000 RPTM) abundant families. Of the 171 miRNA families, five (miR156, miR166, miR1507, miR1509 and miR1510) were categorized as highly abundant. Of these, miR1507 is the most abundant in primary root tips as its expression levels were as high as almost 800,000 RPTM accounting for almost one-twelfth of the total small RNAs sequenced in each of the four libraries. A very high abundance of these 5 miRNA families in this region indicates important gene regulatory roles for these miRNAs in primary root tips. miR156 regulates several SBP transcription factors and miR166 targets HD-ZIP transcription factors whereas miR1507, miR1509 and miR1510 largely found in legumes so far.

Six miRNA families (miR168, miR396, miR1511, miR1508, miR172 and miR3522) were grouped as moderately abundant in primary root tips (Additional file 2). The miR168 family is the most enriched in this group followed by miR396 and miR1511 families. miR168 regulates Argonaute-1, an essential protein for miRNA activity; miR396 regulates several GRF transcription factors that are mostly known to regulate leaf development [17] but also central for normal root growth and development [18] and miR1508 and miR1511 appears to be mostly found in legumes thus far, suggesting lineage-specific roles. miR1507, miR 1508, miR 1509, and miR 1510, are involved in triggering 21 nucleotide phasiRNA biogenesis in legumes [19]. Nineteen miRNA families (miR5767, miR4345, miR169, miR2109, miR482, miR2119, miR2118, miR171, miR164, miR4413, miR390, miR1514, miR159, miR160, miR5781, miR167, miR5373 and miR4409) displayed lower abundances (1000 to 10,000 RPTM) (Additional file 2). This group included a large number of conserved miRNA families, which are known to regulate diverse families of transcription factors such as NF-Y, Scarecrow-like, NAC, MYB, and ARFs. These targets have wide-range of roles regulating diverse developmental as well as other processes in plants [2–4]). Approximately 33 miRNA families (miR1516, miR1517, miR1519, miR1531, miR2107, miR395, miR399 miR4340, miR4350, miR4356, miR4358, miR4368, miR4372, miR4375, miR4382, miR4396, miR4399, miR4401, miR4402, miR4405, miR4414, miR4995, miR5035, miR5369, miR5379, miR5669, miR5673, miR5675, miR5676, miR5679, miR5766, miR5772 and miR862) were extremely low abundant (less than 10 RPTM) in the primary root tips. The extreme low abundance of these miRNAs is in agreement with Arikit et al. [7], who classified these as siRNA-like miRNAs in soybean. Because our classification is strictly based on abundances, this group also included miR395 and miR399, which are highly conserved but expressed at very low levels under normal conditions [20–22].

Among the highly conserved 23 miRNA families [23], two (miR156 and miR166) were classified as high, three (miR168, miR396 and miR172) as moderate and seven (miR169, miR171, miR164, miR390, miR159, miR160 and miR167) as low and two (miR395 and miR399) as extremely low abundantly expressed miRNA families in primary root tips of soybean.

Arikit et al. [7] reported that miR3522 is abundantly expressed in leaf and seed tissues; and miR5770 is enriched in leaf alone. We have identified these two miRNAs in primary root tips (Additional file 2) suggesting that these are ubiquitous in their distribution. It is surprising that this small zone of root tip harbours a diverse population of miRNAs, with 303 miRNAs belonging to 171 families, and several of them are abundantly expressed. These results suggest a critical role for miRNAs in primary root tips, which includes the primary root elongation zone and root apical meristem with actively dividing cells as well as quiescent center (a zone of relatively inactive and slowly dividing cells but can serve as reservoir of stem cells when required) and also the region where vascular differentiation begins. Therefore, it is not surprising to find diverse population of miRNAs that regulates transcription factors as well as other protein coding genes that in turn control multiple developmental processes in the primary root tips.

### Differential expression of miRNA families in the primary roots exposed to water deficit

Most reported plant miRNA regulations in response to stress were based largely on single replicate, whereas we used two replicates. For determining differentially expressed miRNAs during water stress, we used expression differences that are greater than one fold (log2) change and multiple test corrected *P*-values of small than 0.05. In total, 23 miRNA families differentially responded to stress, of which 11 families were upregulated while the other 12 were downregulated (Table 2). Among these, miR1512 was highly upregulated (2.71 log2 fold change) while miR2119 was the most downregulated (4.84 log2 fold change) miRNA families with higher than 100 RPTM in either control or treatment. Interestingly, both of these most differentially regulated miRNAs represent legume-specific miRNA families. Among the conserved miRNAs, miR403 (−1.73 log2 fold change) and miR397 (2.12 log2 fold change) were most differentially regulated families with higher than 100 RPTM in either control or water stress.

**Table 2:** Differentially expressed miRNA families in soybean primary root tips exposed to water deficit

miRNA_family	Control-1 (RPTM)	Control-2 (RPTM)	Ws-1 (RPTM)	Ws-2 (RPTM)	logFC	logCPM	PValue	FDR
MIR5037	70	57	0	0	−9.0	4.1	4.58E-14	1.12E-12
MIR2119	4192	4134	157	133	−4.9	10.1	1.05E-72	9.02E-71
MIR5559	562	434	27	30	−4.1	7.1	1.09E-45	6.21E-44
MIR4408	45	38	7	5	−2.8	3.7	2.11E-04	2.12E-03
MIR1536	60	75	14	15	−2.2	4.4	2.49E-05	2.84E-04
MIR403	104	108	34	30	−1.7	5.2	1.89E-07	2.70E-06
MIR171	3938	3884	1138	1335	−1.7	10.3	4.35E-13	9.30E-12
MIR398	50	57	20	15	−1.6	4.2	2.21E-03	1.40E-02
MIR530	403	434	184	163	−1.3	7.2	2.34E-10	3.64E-09
MIR5370	94	99	34	49	−1.2	5.2	4.08E-04	3.49E-03
MIR4397-3	139	104	41	74	−1.1	5.5	1.68E-03	1.15E-02
MIR4391	462	443	259	177	−1.1	7.4	2.93E-06	3.58E-05
MIR3522	10,815	10,535	24,937	26,151	1.2	13.2	2.33E-07	3.06E-06
MIR408	219	264	504	719	1.3	7.8	5.70E-12	1.08E-10
MIR4344	15	19	55	44	1.5	4.1	5.10E-03	3.01E-02
MIR1535	30	14	68	79	1.7	4.6	1.55E-04	1.66E-03
MIR4411	25	14	82	49	1.7	4.5	1.34E-03	1.01E-02
MIR167	1357	1574	5426	5396	1.9	10.8	6.66E-16	1.90E-14
MIR4385	10	14	48	44	1.9	4.0	2.02E-03	1.33E-02
MIR397	254	174	961	917	2.1	8.2	3.29E-30	1.41E-28
MIR2111	104	80	382	488	2.2	7.1	8.18E-18	2.80E-16
MIR1512	209	207	1459	1281	2.7	8.6	1.57E-79	2.68E-77
MIR4403	0	0	14	30	7.5	2.7	3.69E-04	3.32E-03

Differential regulation of several of these miRNAsin response to water stress was in agreement with previous reports [7–9]. For instance, the observed upregulation of miR397 (stringent criterion) is in agreement with the Kulcheski et al. [9] and downregulation of miR394 (relaxed criterion) (Additional file 2) is similar to the reports of Li et al. [8]. Interestingly, miR169 regulation differs between drought tolerant and drought sensitive soybean genotypes; it is downregulated in a tolerant genotype while upregulated in a sensitive genotype [8, 9]. We used a drought-tolerant soybean cv. Magellan in this study, thus the observed miR169 downregulation (Additional file 2) is consistent with the previous reports [9]. Both of these studies [8, 9] compared the responses in root system while our study specifically analyzed root tips of seedlings.

It was reported that miR1446 is upregulated under drought stress in soybean leaves [7] but this was not even detected in primary root tips. Another observed difference was with respect to miR3522, which was increased in the primary root tips (Table 2 and Additional file 2) but unaffected in leaves [7] in response to stress. On the other hand, the observed upregulation of miR390 as well as downregulation of miR171 in primary root tips (Table 2 and Additional file 2) is similar to what was observed in leaves of soybean exposed to drought [7]. These similar as well as unique responses suggest that miRNA-mediated target gene regulation under drought is similar as well as distinct in leaves and primary root tips of soybean.

We also compared the abundances of individual miRNAs to identify differentially regulated individual miRNAs (with multiple test corrected *P*-values smaller than 0.05), which identified 72 individual miRNAs (Additional file 3). We analyzed the enriched GO terms for 10 of the deregulated individual miRNAs that are highly expressed in either the control or water-stress conditions, and the obtained results were listed in Additional file 4. For example, the targets of an upregulated miRNA, gma-miR156cdeijlm, are enriched in 24 GO terms, such as leaf morphogenesis (GO:0009965) and reproductive developmental process (GO:0003006).

### miR-star abundances in primary root tips and their differential regulation in response to water deficit stress

The processed miRNA duplex include miRNA that is recruited into the Argonaute-1, which is also more stable compared to miR-star, the complementary strand of the duplex. With a few exceptions, the frequency of miR-stars is much lower and largely thought to be non-functional. Consistent with these characteristics, miR-star sequences corresponding to most conserved miRNA families were not recovered from primary root tips (Additional file 1). For those families for which miR-stars were recovered, their abundances were very low compared to their respective miRNA abundances. In the instances where both miRNA and miR-star were sequenced, their normalized abundances did not show any specific ratio. However, some of these miR-star expression levels differed greatly in response to stress, i.e., some were upregulated (miR408a,b,c,d-3p and miR482) while some others were downregulated (miR159d-5p, miR171b-5p, miR172b,h,I,j-5p, miR390a,c-3p, miR394a-3p, and miR1507c-5p) (Additional file 1).

### Novel miRNA identification

**Fig. 2:**
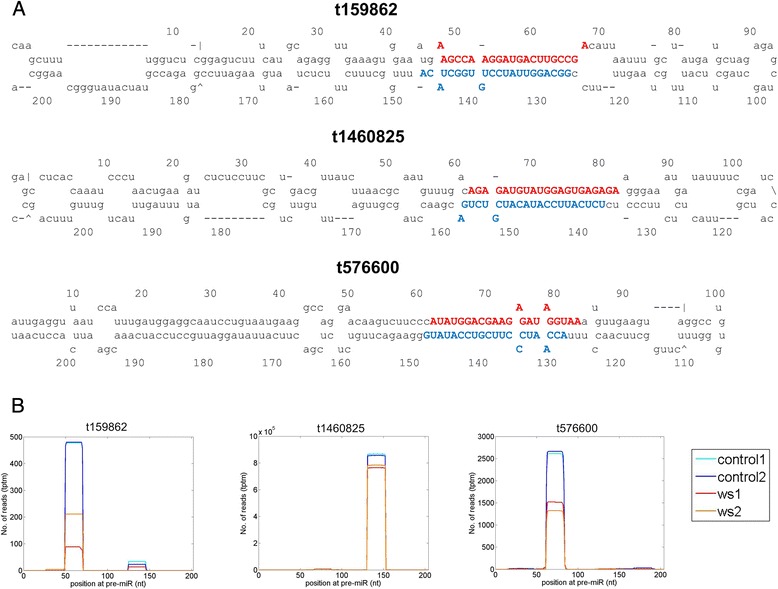
Some of the novel miRNAs in soybean. **a** The secondary structures of six novel miRNAs. *Red* and *blue* regions are mature miRNAs detected in the sequencing libraries. **b** The distributions of small RNA reads on the novel pre-miRNAs in Part a

Several deep sequencing efforts have identified many novel miRNAs in soybean [7–9]. Because we used a very specific tissue (primary root tips) in this study, we scrutinized the small RNAs for novel miRNAs, if any. Indeed, on the basis of sequencing both miRNA and miRNA-star sequences [13], we have identified 22 novel miRNAs in the sequenced libraries (Additional file 5). As shown in Fig. 2a, the miRNA and its miRNA* reads were detected for novel miRNAs. Figure 2b shows that miRNAs generated from these pre-miRNAs are from either 5′ or 3′ arms of the hairpin structures. Other novel miRNAs in Additional file 4 are similar to the three examples shown in Fig. 2. Only one novel miRNA is significantly downregulated in water-stressed libraries (multiple test corrected *P* = 0.004, in Additional file 6).

## Discussion

Prolonged exposure to drought affects plant growth and development, consequently crop yields; even transient droughts can affect plant productivity significantly [24]. Different layers of gene regulatory mechanisms are critical for enduring drought tolerance in plants and miRNA-dependent post-transcriptional gene regulation is the most recent addition to this list. Primary root tips are critical zone of a root system for water uptake as well as water stress sensing and signalling. We profiled miRNAs in response to water stress in soybean to gain an insight into miRNA-guided gene regulations and its role in stress adaptation. Using stringent criterion, we have identified 12 families (6 families were each up- or down-regulated) as differentially regulated during drought stress (Table 2). However, using relaxed criterion, additional 12 miRNA families were also identified as differentially regulated. Overall, 24 families were found to be differentially expressed in response to water stress in soybean primary root tips (Table 2).

By regulating the expression of SBP transcription factors in leaves, miR156 is known to regulate plant progression into reproductive phase from vegetative phase [25–27]. Besides in leaves, miR156 is abundantly expressed in primary root tips (this study) and the nodules [7] suggesting a much broader role for miR156 family in plants. In different plant species, miR156 is largely upregulated under drought [5]. Most recently, Stief et al. [28] demonstrated that miR156 plays an important role in heat tolerance of Arabidopsis. Even in their study, only 3 variants (MIR156h, d and c) of miR156 family were found to respond to heat suggesting that the overall miR156 regulation would only be marginal and yet sufficient to make Arabidopsis plants to adapt to the recurrent heat by affecting the SBP factors expression [28]. Of the seven miR156 variants we identified in the primary root tips, four are abundantly expressed and were unaffected by the treatment, while two variants that are expressed at low levels (miR156b/f and miR156r) are highly upregulated under stress (Additional file 1). This observation suggests a role for specific variants of miR156 under water stress, probably in specific cell types within the primary root tip.

Being negative regulators of their target mRNAs, downregulation of a miRNA under stress could result in upregulation of the targets and the opposite is true for the upregulated miRNAs [5]. The regulation of transcription factors was studied under water stress conditions in five-centimeter long root primary roots of soybean [12]. Most importantly, the study found the transcription factors (two CBF-B/NF-YA, a MYB33 gene and TEM1, TEMPRANILLO 1) [7] that are targets of miRNAs were differentially regulated under drought [12]. Our miRNA analysis revealed that the miRNAs targeting above mentioned transcription factors were differentially regulated; miR482 was upregulated while miR169 was downregulated below the stringent threshold level of(log2) fold change (Additional file 2). Our results are consistent with the reported upregulation of two NF-YA genes (Glyma02g47380 and Glyma10g10240) [12] that are targeted by miR169 and decreased expression of TEM1 transcription factor [12] that is targeted by miR482. These results demonstrate that the miRNA regulations affected their target genes under stress in the soybean primary root tips.

In soybean primary roots, 37 transcripts associated with auxin synthesis and signaling were regulated by drought [12]. Of these, 17 are auxin response factors that include targets of miR167 (Glyma16g02760.1) and TAS3siRNAs (Glyma06g00950.1), which are downregulated especially under mild and severe stress conditions [12]. The downregulation of these 2 ARFs (Glyma16g02760.1 and Glyma06g00950.1) in primary root tips is consistent with the observed upregulation of miR167, which is induced by almost 2 fold (Table 2). The moderate upregulation of miR390 under water deficit stress could lead to the enhanced accumulation of TAS3 siRNAs as observed in the present study (Additional file 1), which in turn decreases the accumulation of ARFs (ARF2, ARF3 and ARF4). Similarly, the levels of miR393 were also elevated under stress and miR393 targets TIR1, an auxin receptor in plants. The elevated miR393 could decrease TIR1 levels under stress and this will results in decreased ubiquitination of AUX/IAA factors that act as transcriptional repressors. This is consistent with the hypothesis that miRNAs play a critical role in auxin-dependent growth modulation under stress [5]. Overall, three miRNA families that are associated (miR167, miR393 and miR390 and TAS3 siRNAs all displayed either high or moderate upregulation) with auxin sensing and signalling are upregulated in response to water stress. Such a coordinated differential regulation of miRNAs and TAS3siRNAs that target auxin sensing and signaling suggests a more compelling role for these small RNAs under stress. This is somewhat consistent with the fact that the decelerated growth rate in the Region 2 of the primary root tips under drought could be mediated by three miRNAs for conserving water and energy.

miR160 is the only other miRNA family that is known to be involved in auxin signalling but unaltered in response to water stress in this study. In fact, miR160 targets ARF10, ARF16 and ARF17, which have been characterized as transcriptional repressors. Further studies are required to determine whether or not miR160 as well as the other miRNAs that control auxin signaling are differentially regulated under drought in different regions (Region1 and Region 2) of the primary root tips of soybean.

The auxin-mediated gene regulation is dependent on ARFs, but their exact roles as positive or negative regulators of gene expression are largely unknown. On the basis of Arabidopsis ARF promoter:GUS analysis in protoplasts, some have been characterized as activators while some other as repressors [29]. Thus, the overall in vivo role of these ARFs is still unclear in plants.

In Arabidopsis, Cu/Zn SOD upregulation in response to oxidative stress is exclusively dependent on the downregulation of miR398 [5, 20]. miR398 in plants controls Cu/Zn SOD protein abundances not only at the posttranscriptional level by targeting Cu/Zn SOD transcripts but also at the posttranslational level by targeting the CCS gene, that supplies Cu to the Cu/Zn SODs in plants [5, 30]. Indeed miR398 levels were down-regulated under water stress in primary root tips of soybean (Table 2). These results are consistent with previous reports that the protective proteins associated with the ROS toxicity were upregulated in this region of soybean primary roots exposed to water stress [10].

Recently, several studies reported differential expression of miRNAs such as miR164, miR169, miR171, miR396, miR398, miR399, miR408 and miR2118 in drought-stressed plants but their regulation (up or down) differed between different plant species [5]. Previously, Li et al. [8] analyzed changes in miRNA profiles in roots of a drought-sensitive soybean genotype (inbred line ‘HJ-1’) while Kulcheski has compared between drought-sensitive ‘BR16’ with a drought-tolerant ‘Embarpa 48’. Li et al. [8] exposed 4 week-old hydroponically grown seedlings to 2% PEG in 1x Hoagland’s solution for 48 h, while Kulcheski et al. [9] studied in 15 day-old seedlings subjected to drought stress by removing the hydroponic solution (similar to that of air drying) for 125 and 150 min. These studies found similar as well as distinct regulations with respect to the responses of various conserved miRNAs. For instance, Li et al. [8] found that miR166b, miR169d, miR1507a, miR167, miR482, miR4369 and miR4397 were upregulated while miR394 is downregulated. On the other hand Kulcheski et al. [9] have reported that miR166-5p, miR169f-3p, miR1513c, and miR397a,b, were upregulated in sensitive genotype BR16 while these were upregulated in tolerant Embarpa48. Such discrepancies could be due to differences in genotypes, treatments, duration or other variables. Interestingly, miR169 regulation differs between drought tolerant and drought sensitive soybean genotypes; it is downregulated in a tolerant genotype while upregulated in a sensitive genotype [8, 9]. We used a tolerant soybean cv. Magellan in this study, thus the observed miR169 downregulation is consistent (Additional file 2) with the previous reports [9]. Both of these studies [8, 9] compared the responses in an entire root system while our study specifically analyzed root tips of seedlings. Differential responses of miRNAs could be due to several factors such as whether plant species is stress sensitive or tolerant, the tissue that was analyzed, the severity and duration of stress treatment as well as the fact the some of the miRNAs respond transiently while some others show sustained responses to a given stress [31].

To gain an insight into miRNA regulations that are common to diverse abiotic stresses or specific to water-deficit, we compared our results with Pi and N-deprivation responses that are available for root comparisons in soybean. Under Pi deficiency downregulation of miR169 and miR159 whereas upregulation of miR482 and miR397 was reported [32] and similar responses were found during water deficit (Table 2 and Additional file 2). However, miR3522 differed in its response that it was downregulated under Pi deficiency [32] while it was upregulated in our study. Another study [33] reported upregulation of miR159 under Pi deficiency in soybean roots, which contradicts with the results from Xu et al. [32] as well as our analysis under water deficit stress (Additional file 2 and Table 2). In response to N deprivation, down regulation of miR171 and miR398 [34] was reported, which is in agreement with our analysis under water stress. On the other hand, miR166 and miR390 was downregulated by N deficiency [34] whereas these were upregulated under water stress (Additional file 2 and Table 2). Overall it appears that there are similarities and differences with respect to miRNA regulations under water stress and nutrient deprivations. However, it should be noted that our study used primary root tips while those compared studies used entire root system, so these comparisons need to be cautiously interpreted.

## Conclusions

Both root elongation and differentiation processes are important aspects of how plants respond to a variety of environmental factors that limit growth. Characterization of roles of miRNAs in stress regulatory networks in the primary root tips would help better understand the mechanisms of stress tolerance as well as provide new opportunities to engineer plant’s drought tolerance. Yamaguchi et al., [10] had investigated root growth adaptation to low water potential in soybean, specifically with respect to the protein alterations in the elongation zone of the primary root tip (region-specific analysis). To maintain root elongation under water deficit, two potential mechanisms have been suggested: osmotic adjustment and modification of CW extensibility [35]. Osmotic adjustment mostly involves net accumulation of osmolytes such as sugars (fructose, glucose, and sucrose), amino acids (mostly proline) and glycine betaine [24, 36]. Modification of cell wall extensibility mostly includes protein synthesis and modification of carbohydrates. None of the differentially regulated miRNAs can be directly linked with such processes. However, it is worth noting that TFs targeted by miRNAs could be regulating many genes that control these two major processes, thus, miRNAs are likely to play an indirect role in modulating drought responses in the primary root tips.

## Additional files

Additional file 1: Table S1. The normalized abundances of individual conserved miRNAs. (XLSX 46 kb)
Additional file 2: Table S2. The normalized abundances of miRNA families in control and water-stressed roots tips of soybean. (XLSX 36.2 kb)
Additional file 3: Table S3. The deregulated miRNAs in water-stress condition. (XLSX 19 kb)
Additional file 4: Table S4. The enriched GO terms of predicted targets for ten deregulated miRNAs in water-stress conditions. (XLSX 149 kb)
Additional file 5: Table S5. The novel miRNAs identified in soybean. (XLSX 14 kb)
Additional file 6: Table S6. The normalized abundances of novel miRNAs. (XLSX 13 kb)
